# Lignin engineering through laccase modification: a promising field for energy plant improvement

**DOI:** 10.1186/s13068-015-0331-y

**Published:** 2015-09-15

**Authors:** Jinhui Wang, Juanjuan Feng, Weitao Jia, Sandra Chang, Shizhong Li, Yinxin Li

**Affiliations:** Key Laboratory of Plant Molecular Physiology, Institute of Botany, Chinese Academy of Sciences, Beijing, 100093 China; Beijing Engineering Research Center for Biofuels, Tsinghua University, Beijing, 100084 China; Institute of Nuclear and New Energy Technology, Tsinghua University, Beijing, 100084 China

**Keywords:** Laccase, Lignin, Lignocellulose, Biofuel, Genetic engineering

## Abstract

Laccase (*p*-diphenol:dioxygen oxidoreductase, EC 1.10.3.2) is a member of the multicopper oxidases and catalyzes the one-electron oxidation of a wide range of substrates, coupled with the reduction of oxygen to water. It is widely distributed in bacteria, fungi, plants and insects. Laccases are encoded by multigene family, and have been characterized mostly from fungi till now, with abundant industrial applications in pulp and paper, textile, food industries, organic synthesis, bioremediation and nanobiotechnology, while limited researches have been performed in plants, and no application has been reported. Plant laccases share the common molecular architecture and reaction mechanism with fungal ones, despite of difference in redox potential and pH optima. Plant laccases are implicated in lignin biosynthesis since genetic evidence was derived from the Arabidopsis *LAC4* and *LAC17*. Manipulation of plant laccases has been considered as a promising and innovative strategy in plant biomass engineering for desirable lignin content and/or composition, since lignin is the major recalcitrant component to saccharification in biofuel production from lignocellulose, and therefore directly limits the fermentation yields. Moreover, plant laccases have been reported to be involved in wound healing, maintenance of cell wall structure and integrity, and plant responses to environmental stresses. Here, we summarize the properties and functions of plant laccase, and discuss the potential of biotechnological application, thus providing a new insight into plant laccase, an old enzyme with a promising beginning in lignocellulose biofuel production.

**Electronic supplementary material:**

The online version of this article (doi:10.1186/s13068-015-0331-y) contains supplementary material, which is available to authorized users.

## Background

As a renewable, environmental-friendly and economical resource, biofuel has received much attention in recent years. After the “first generation” biofuel derived from starch and sugar-based raw materials like maize, sugarcane and sugar beet, the “second generation” biofuel production based on lignocellulosic biomass is emerging as a new promising industry [1]. Generally, the process of the second generation biofuel production includes pretreatment for liberation of polysaccharides, release of monomeric sugars based on enzymatic hydrolysis, fermentation and subsequential distillation. Feedstock for lignocellulosic biofuel production can be non-edible energy crops (Miscanthus, switchgrass, sweet sorghum, etc.), agricultural and forest residues, as well as municipal and industrial wastes, which are usually economically feasible, noncompeting with food supplies, and can be produced in large quantities [2].

The major component of the lignocellulosic biomass is the plant cell wall, a heterogeneous complex mainly consisting of cellulose, hemicellulose and lignin [1]. In the conversion process, lignin has been identified as the primary recalcitrant component to saccharification, since it tends to adsorb cellulolytic enzymes and restrict cellulose release, also the degradation byproducts can inhibit the activity of cellulolytic enzymes [3, 4]. As lignin directly limits the fermentation yield, its removal by pretreatment or modification prior to enzymatic hydrolysis and fermentation would be advantageous. Genetic engineering of plant cell wall has been considered as a promising strategy for desirable lignin content and/or composition towards higher biofuel production [5]. Manipulation of laccase, which catalyzes monolignols oxidation and polymerization in plant lignin synthesis, would be a great approach for plant biomass engineering [6, 7].

Laccase (*p*-diphenol:dioxygen oxidoreductase, EC 1.10.3.2), a family of copper-containing polyphenol oxidases, belongs to the multicopper oxidases (MCOs). Though first identified in the exudates of the Japanese lacquer tree *Rhus vernicifera*, studies on laccase have been performed predominantly in fungi in the past decades. And a great many reviews concerning fungal laccase have been published with emphasis on the general characteristics [8–13], catalytic properties [14–16], biological functions [17–19], industrial applications [20–26], and laccase engineering for higher catalytic activity and stability [27–29]. Compared to the extensive studies on fungal laccases, the research of plant laccases is just emerging in the past several years. This paper presents an overview of the current knowledge of plant laccases in aspects of the essential characteristics, gene expression pattern and regulation, genetic view of plant laccases in lignin biosynthesis, and divergent functions. Finally, the potential applications of plant laccase in energy plant engineering and phytoremediation are discussed.

## Laccase: different sources with common structure but distinct properties

**Fig. 1:**
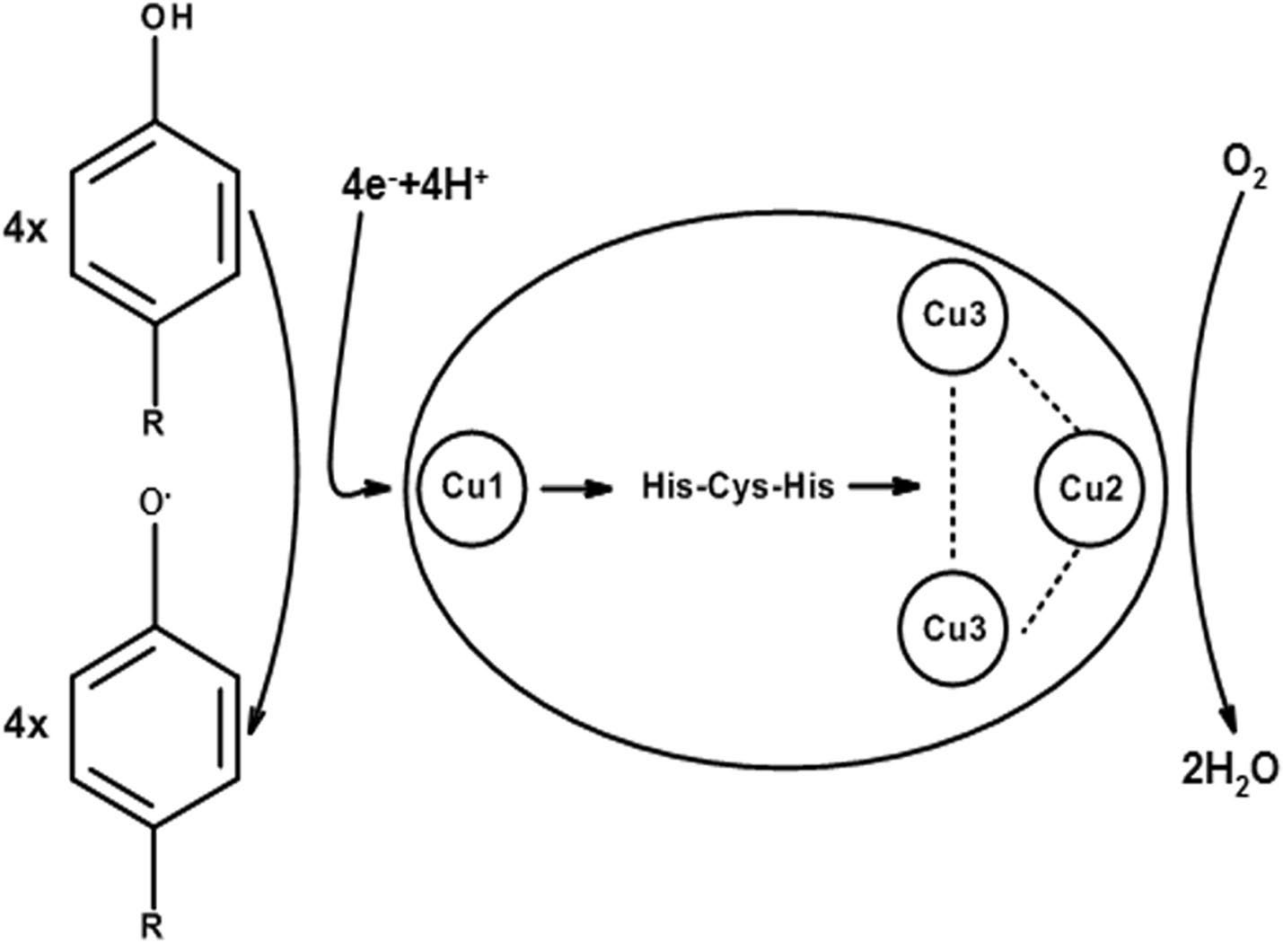
Schematic diagram of laccase catalytic cycle, adapted from Baldrian [10]. The laccase substrate is oxidized by Cu1, involving the loss of a single electron and the formation of a free radical, which may undergo further oxidation or nonenzymatic hydration, disproportionation and polymerization. Reduction of Cu1 is rate-limiting in the catalytic process. The extracted electron is transferred through highly conserved His-Cys-His motif to the trinuclear copper cluster, where molecular oxygen is reduced to water. The reduction of one molecule of oxygen to two molecules of water is accompanied by oxidation of four molecules of substrate

Typically, laccase contains four copper ions classified by different spectral and electronic paramagnetic resonance (EPR) properties: a mononuclear “blue” copper ion (Cu1) at the T1 site which confers the typical blue color to the protein, and a trinuclear copper cluster at the T2/T3 site, consisting of one T2 copper ion (Cu2) and two T3 copper ions (Cu3) [8, 9, 30]. Each of the copper ions plays a dissimilar role in the one-electron oxidation of various substrates, such as *ortho*- and *para*-diphenols, aminophenols, polyphenols, polyamines and aryl diamines, concomitantly with the full reduction of molecular oxygen to water (Fig. 1) [14, 25, 31]. Specifically, T1 is the site where substrate oxidation takes place while the reduction of oxygen and release of water occurs at the T2/T3 site [32, 33].

Laccase is widely distributed in plants, insects and microbes (including bacteria and fungi), and is encoded by multigene family, for example, the model plant Arabidopsis genome encodes 17 laccases, and the number of identified laccase genes in the *Trametes villosa* fungus and the insect *Nephotettix cincticeps* is 5 and 3, respectively [34–36]. Fungal laccases have been comprehensively identified from ascomycetes, deuteromycetes, and basidiomycetes especially the white-rot basidiomycetes, and are involved in lignin degradation, morphogenesis, pathogenesis and stress defence [8–10]. Genomic screening in bacteria has found widespread occurrence of laccases as well, although sequence and structure characterization were not widely reported [37–39]. The best-studied bacteria laccase so far is CotA in *Bacillus subtilis*, which participates in pigment biosynthesis and protection against UV light [40]. Moreover, laccase has been identified in archaea, such as the LccA from halophilic archaeon *Haloferax volcanii*, a highly thermostable and salt/solvent-tolerant laccase [21, 41]. In addition to microbial laccases, insect laccases have been continually identified in *Monochamus alternatus*, *Bombyx mori*, *Apis mellifera*, etc., which are mainly associated with cuticle sclerotization of insects [39, 42–44]. And to date, laccases have also been widely identified from plants besides Anacardiaceae, such as loblolly pine (*Pinus taeda*), sycamore maple (*Acer pseudoplatanus*), tobacco (*Nicotiana tabacum*), poplar (*Populus trichocarpa*), yellow poplar (*Liridendron tulipifera*), ryegrass (*Lolium perenne*) [45–50], etc. Moreover, in-depth research has been reported in *Arabidopsis thaliana*, maize (*Zea mays*), rice (*Oryza Sativa*), sugarcane (*Saccharum officinarum*), *Brassica napus* and *Brachypodium distachyon* [7, 51–55].

**Fig. 2:**
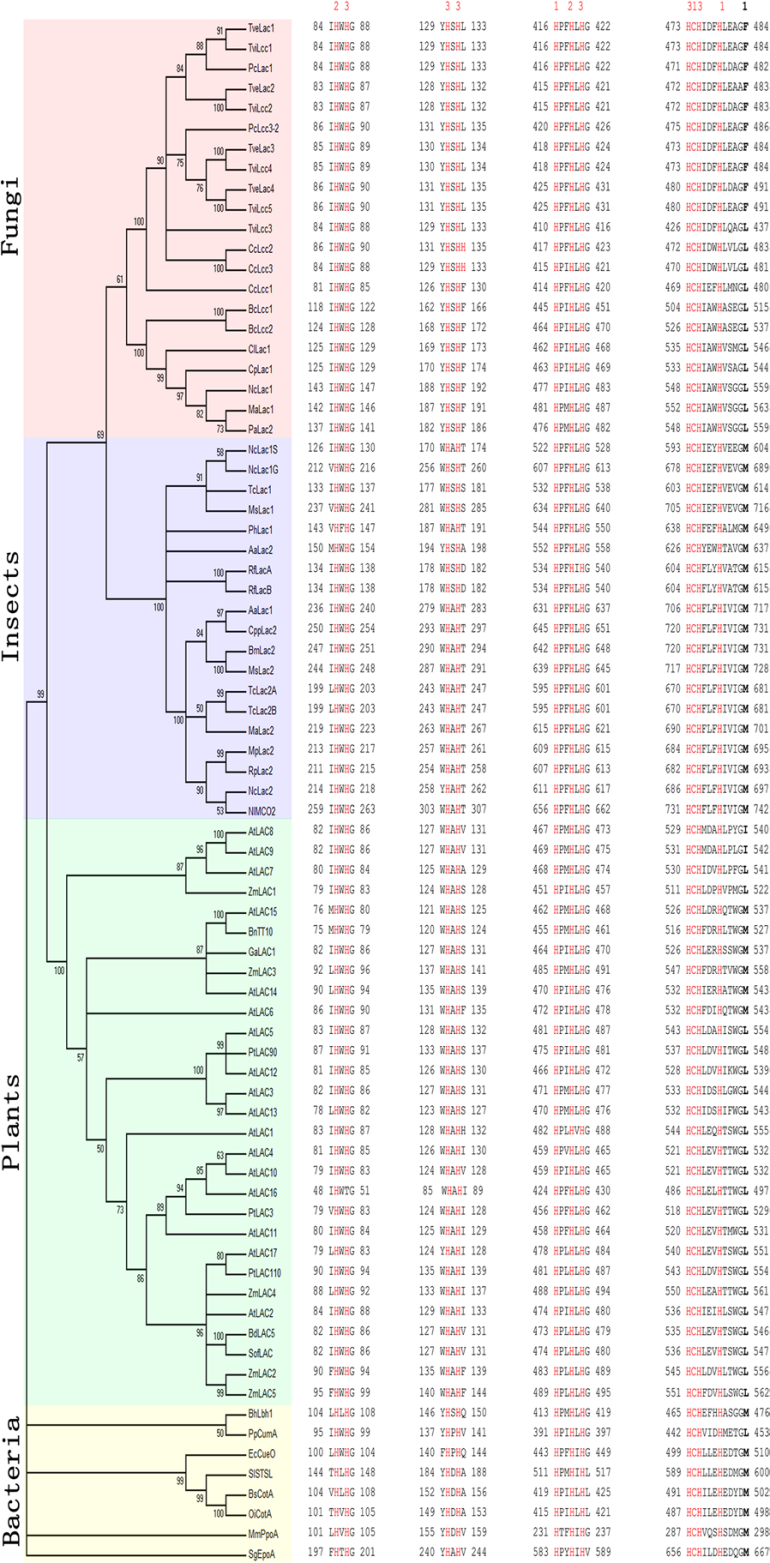
Phylogenetic analysis of representative laccases from plants, fungi, insects and bacteria. Laccases are encoded by multigene family, for example, the Arabidopsis genome encodes 17 laccases, and the number of identified laccase genes in *Trametes villosa* and *Nephotettix cincticeps* is 5 and 3, respectively. Laccases from plants, fungi, insects and bacteria are clustered separately in the phylogenetic tree for each taxon, but share conserved copper binding sites. The amino acids potentially involved in copper binding include ten histidines and one cysteine (in *red*), with numbers 1, 2 and 3 corresponding to the Cu1, Cu2 and Cu3 ions. An axial ligand of methionine, leucine, or phenylalanine (in *bold*) was indicated. The bootstrap consensus tree was constructed by MEGA with the Neighbor-Joining method. Database accession numbers of laccase sequences are listed in Additional file 1: Table S1

Laccases from plants, fungi, insects and bacteria are clustered separately in phylogenetic tree, although all of them have conserved copper binding amino acids, including ten histidines, one cysteine and an axial ligand of methionine, leucine or phenylalanine (Fig. 2). The axial ligand can roughly indicate the redox potential of a given laccase: a coordinating methionine for low potential whereas a non-coordinating leucine or phenylalanine for middle and high potential, respectively, but there are exceptions [14, 30, 56]. Generally, plant and insect laccases have lower redox potential. Microbial laccases fall into all three groups, among which the bacteria laccases belong to the low-redox-potential group, the basidiomycete white-rot fungi laccases mainly constitute the high-redox-potential group, and the majority of ascomycete and some basidiomycete fungi laccases are attached to the middle-redox-potential group [27, 29].

## General enzymology characteristics of plant laccase

**Fig. 3:**
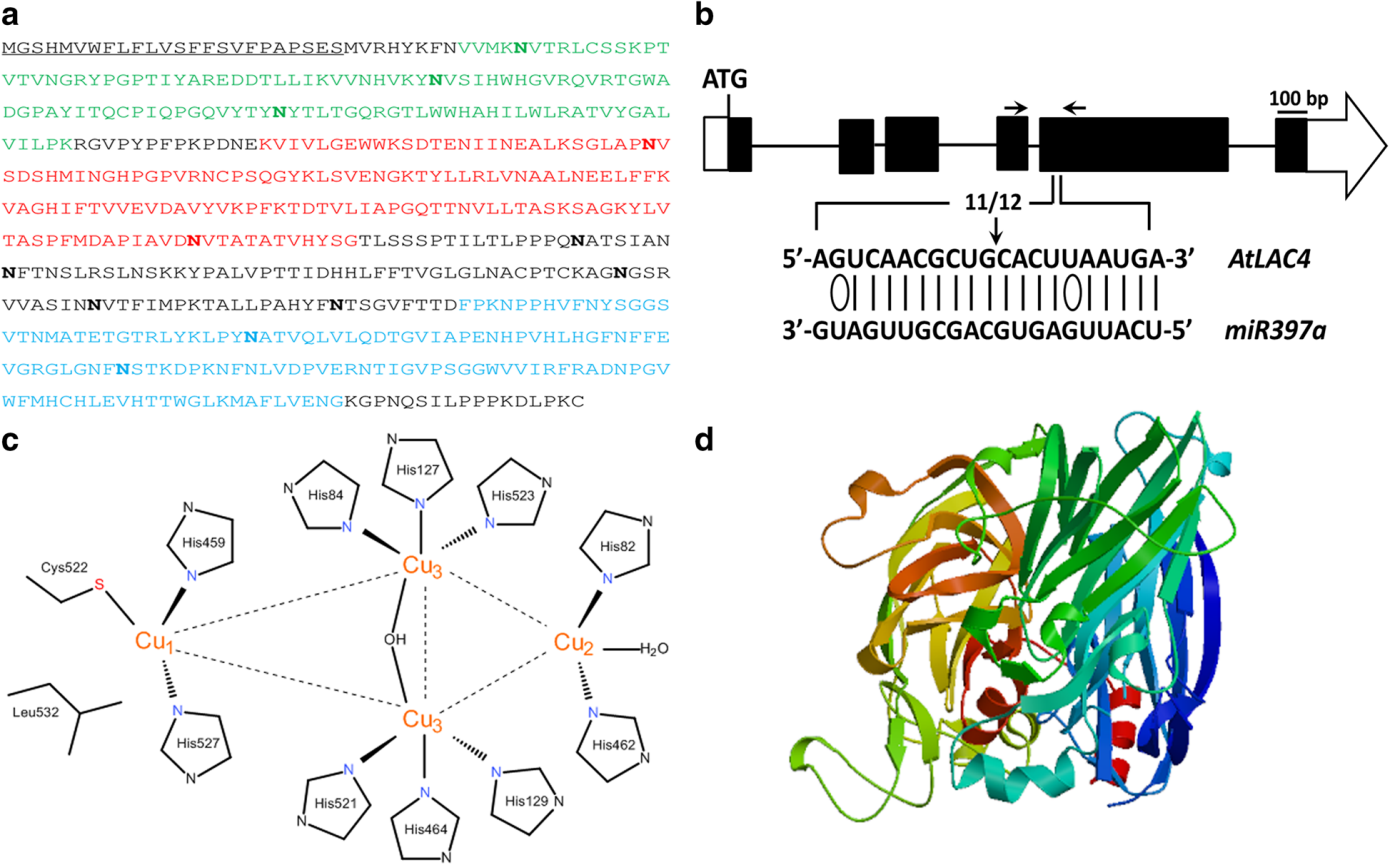
Sequence and structure of AtLAC4 from *A. thalina*. **a** The amino acid sequence of AtLAC4. The protein consists of 558 amino acids and weighs 61.5 kDa, with theoretical pI value of 9.31. It has a signal peptide (*underlined*) at the N-terminal predicted by SignalP 4.1 Server [98] and three conserved Cu-oxidase domains (colored in *red*, *blue* and *green*, respectively), according to Pfam [99]. Twelve asparagines predicted to be N-glycosylated by NetNGlyc 1.0 Server [100] were indicated in *bold*. **b** *MiR397a* mediated cleavage of *AtLAC4*, adapted from Abdel-Ghany et al. [63]. The *black boxes* represent exons and the *horizontal lines* represent introns. The *white box* represents 5′-UTR, while the *white arrow* represents 3′-UTR. *Vertical arrow* indicates the 5′ termini of miRNA-guided cleavage products, with the frequency of clones shown. Watson–Crick pairing (*solid lines*) and G:U wobble pairing (*ellipse*) between *AtLAC4* target sequence and the complementary *miRNA397a* sequence are indicated. **c** The view of ligands at the copper center of AtLAC4. Cu1 is coordinated with two histidines, one cysteine and one leucine, Cu2 is coordinated by another two histidines and one H_2_O ligand, while six histidines coordinate the Cu3 pair in a symmetrical manner, with a bridging OH ligand. **d** Three-dimensional structure of AtLAC4 predicted by SWISS-MODEL [101]

Plant laccases are glycoproteins with higher carbohydrate content (20–45 %) than fungal laccases (10–25 %), which has been reported to be responsible for copper retention, enzyme stability and activity. The molecules usually consist of 500–600 amino acids and weigh approximately 60–130 kDa, while the isoelectric point (pI) values range from 7.0 to 9.6 (Fig. 3a) [8, 56]. Most plant laccases are secreted proteins, with a few exceptions predicted to be located in mitochondria.

Although, the active sites of plant laccases share the common molecular architecture (Fig. 3d) and reaction mechanism with fungal ones, they have lower redox potential and different pH requirements. The lower redox potential is due to a methionine or leucine rather than phenylalanine residue at the T1 site of plant laccases (Figs. 2, 3c) [9]. The pH optima greatly differs between laccases of plant and fungus origin, as the optimal pH for plant laccases is around physiological range of 7.0–10.0 while a lower acidic pH is optimal for the fungal ones [57]. Additionally, altered microenvironment at the active site was also predicted for both of the laccases [58, 59]. These characteristics may partly account for the different functions of plant and fungal laccase, one catalyzes lignin biosynthesis while the other is responsible for degradation, respectively. Further research is required to distinguish other differences.

## Regulation of expression and subcellular localization of plant laccase

Plant laccases are expressed diversely in different tissues at various developmental stages. Tissue-specific expression patterns have been reported in Arabidopsis, such as *LAC4* specifically expressed in interfascicular fibers, vascular bundles and seed coat columella, *LAC7* in hydathodes and root hairs, *LAC8* in pollen grains and phloem, *LAC15* in seed coat cell walls, and *LAC17* in interfascicular fibers [34, 60]. And in sugarcane, *SofLAC* has been detected to be preferentially expressed in sclerenchymatic bundles and parenchymatic cells surrounding the vascular bundles of young internodes [51]. The tissue-specific expression profiles were also detected for eight of the *P. taeda* laccases [61].

There are multiple *cis*-elements in promoter sequences of Arabidopsis laccases, indicating potential roles of transcription factors in regulating laccase expression [34]. MYB58, for instance, one of the SND1-regulated MYB transcription factors, is able to activate the expression of *LAC4* gene directly [62]. In addition, 15 of the 17 laccases from Arabidopsis (except *LAC6* and *LAC14*) contain copper response elements in promoter regions, suggesting the expression of these genes may be responsive to Cu levels [34]. Recently, miRNAs have also been reported to target laccases and function in the regulation of laccase expression at post-transcriptional level. Several laccases were potentially regulated by miRNAs under Cu deficient conditions in Arabidopsis, among which miR408 was predicted to target the coding sequences of *LAC3*, *LAC12* and the 5′-UTR of *LAC13*; miR397 was predicted to target the fifth exon of *LAC2*, *LAC4* (Fig. 3b) and *LAC17*; while miR857 was predicted to target the first exon of *LAC7*. Furthermore, the expression of these miRNAs was negatively correlated with that of the laccase targets [63]. A recent study found the involvement of miR397b in regulating both lignin content and seed number in Arabidopsis via modulating *LAC4* [6]. Similar mechanisms have also been revealed in *P. trichocarpa* and *O. Sativa*, where Ptr-miR397a and OsmiR397 perform as negative regulators of *PtrLACs* and *OsLAC*, respectively [7, 64].

The expression of certain plant laccases are responsive to environmental stresses, based on large-scale sequencing analysis or expression detection. For example, the transcript level of a laccase gene was induced in tomato roots treated with 170 mM NaCl [65]. Similar increase of *ZmLAC1* transcripts was observed in maize primary roots treated with varied NaCl concentrations [52]. Another study also detected sharp increase of laccase transcripts responding to lead (Pb) stress in maize, which was suggested to account in part for Pb hyperaccumulation in the line 178 [66].

Most plant laccases are predicted to be localized in the apoplast, on account of the presence of N-terminal signal peptide (Fig. 3a) which directs the protein into the secretory pathway. Though, limited experimental data have been gathered regarding the subcellular location of plant laccases. It has been reported in Arabidopsis that *LAC4* and *LAC17* are located in the secondary cell walls throughout protoxylem tracheary element differentiation [67]. And in *B. distachyon*, *BdLAC5* and *BdLAC6* were detected in the apoplasm in lignified interfascicular fibers [55]. The exception worth noting is that *AtLAC15* was reported to be observed within the vacuole lumen instead of the cell wall, which may be related to its function [68].

## Genetic evidence for the involvement of plant laccase in lignin biosynthesis

**Fig. 4:**
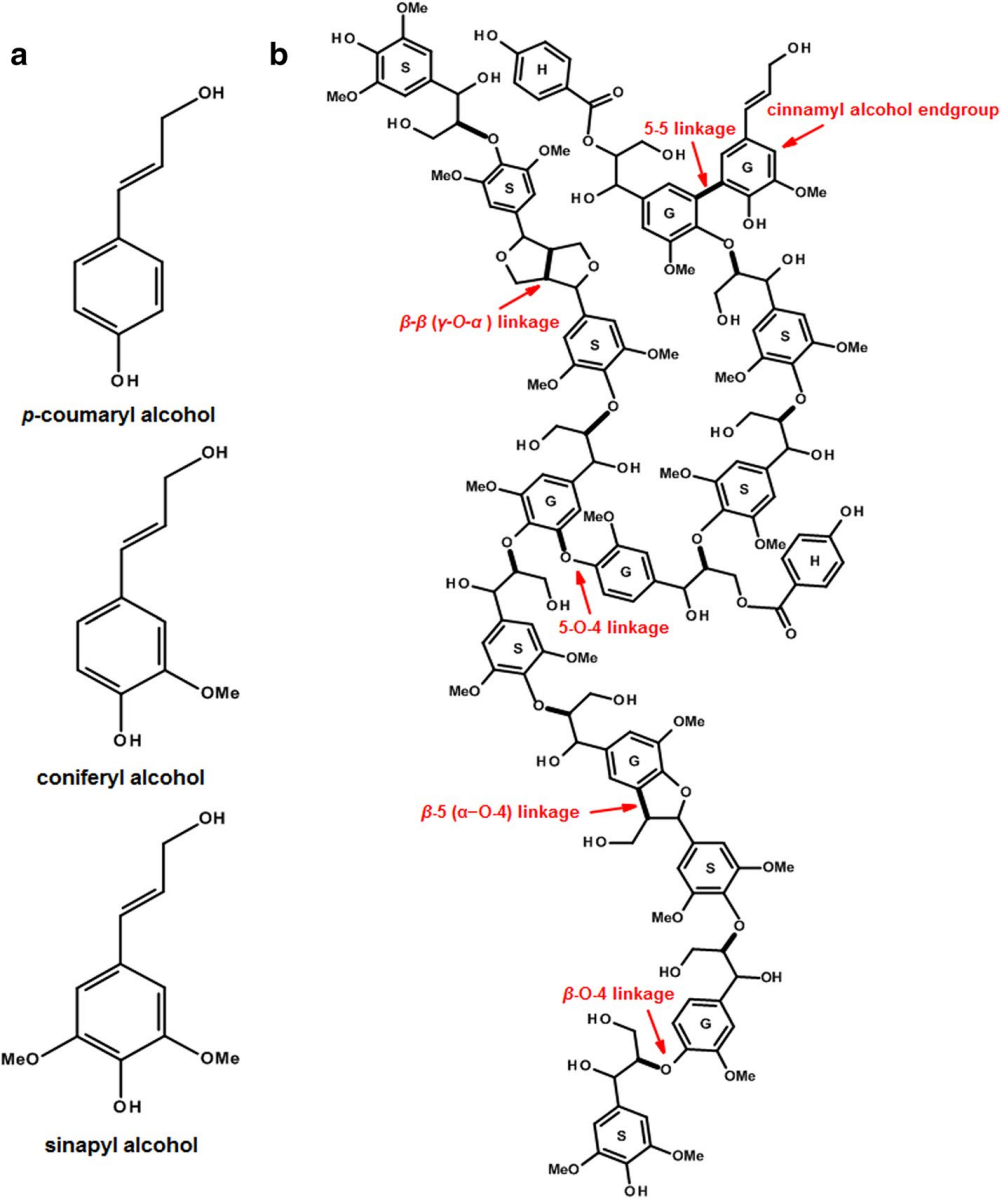
Monolignols and a lignin polymer model. **a** Primary lignin monomers: *p*-coumaryl alcohol, coniferyl alcohol and sinapyl alcohol. The three monolignols differ in the degree of methoxylation, and are catalyzed by peroxidase and/or laccases to form the corresponding *p*-hydroxyphenyl (H), guaiacyl (G), and syringyl (S) lignin unit, respectively. **b** A polymer model depicting the general feature of lignin. The model is constructed by Accelrys Draw 4.2 with two H units, six G units and eight S units, showing the main linkage types. The *bolded bonds* are formed in the radical coupling reactions. This is only a model, and do not imply any primary structure or composition of plant lignin

Being contrary to the ligninolytic activity of fungal laccases, plant laccases are involved in lignin polymerization [69, 70]. Lignin, the second most abundant biopolymer after cellulose, accounts for approximately 30 % of the organic carbon in the biosphere and has been essential in the evolutionary adaptation of plants from aquatic environment to land [71]. Lignin is derived primarily from three hydroxycinnamyl alcohol monomers: coniferyl, *p*-coumaryl and sinapyl alcohols (termed monolignols) (Fig. 4a), which are synthesized in the cytosol and thereafter is exported into the cell wall for endwise polymerization and incorporation into guaiacyl (G), *p*-hydroxyphenyl (H) and syringyl (S) lignin units, respectively (Fig. 4b) [72, 73]. The oxidation of monolignol molecules is thought to be catalyzed by one or more peroxidases; however, increasing evidences have indicated the involvement of laccase in this process [74, 75].

In vitro experiments were performed in *P. taeda* and *A. pseudoplatanus* as early as 20 years ago to associate laccase with lignification [45, 76]. Later in *P. trichocarpa*, three independent antisense poplar lines, *lac3*AS, *lac90*AS, and *lac110*AS were generated, but no significant alteration in lignin content and composition was observed. However, *lac3*AS exhibited a two- to threefold increase in total soluble phenolic content and a dramatic alteration of xylem fiber cell walls. The results indicated that *LAC3* is essential for normal cell wall structure and integrity of poplar xylem fibers [77]. Chemical component analysis of the Arabidopsis *lac15* mutant seeds revealed nearly 30 % decrease in extractable lignin content compared with wild-type seeds [78]. This was the first direct evidence for possible role of laccase in lignin synthesis.

Genetic evidence was not achieved until the year 2011 when Berthet et al. produced two double mutants of *lac4*-*1lac17* and *lac4*-*2lac17*, the lignin content of which were 20 and 40 % lower than those of the control, respectively, while the single mutants *lac4* and *lac17* had moderately reduced lignin levels. Complementation of *lac17* with *LAC17* restored the mutant to a normal lignin profile. This study provided the first in vivo evidence that both of *LAC4* and *LAC17* contribute to the constitutive lignification of Arabidopsis stems [60]. Moreover, simultaneous disruption of *LAC11* along with *LAC4* and *LAC17* almost completely abolished lignin deposition in *lac4lac11lac17* triple mutant, causing severe plant growth defect, whereas the casparian strip develops normally through the activity of peroxidase, suggesting that laccase is necessary and nonredundant with peroxidase for lignin polymerization during vascular development in Arabidopsis [79]. Another research in sugarcane discovered that the expression of *SofLAC* restored the lignin content but not the lignin composition in complemented Arabidopsis *lac17* mutant, suggesting that *SofLAC* participates in lignification in sugarcane [51]. These findings indicate that genetic engineering of lignin-specific laccases is a potentially innovative and promising tool for fine-tuning lignin content and/or composition.

## Diverse functions of plant laccase

Plant laccases are also involved in varieties of biological processes such as wound healing [17, 80], iron metabolism [81], and maintenance of cell wall structure and integrity [77]. It has been reported that mutants of the Arabidopsis laccases exhibit multiple phenotypes, suggesting different roles performed by them. For example, *lac2* showed compromised root elongation under PEG-induced dehydration conditions; *lac8* flowered earlier than wild-type plants, and *lac15* showed an altered seed color [82].

Certain laccase members are predicted to take part in the polymerization of phenolic compounds. The Arabidopsis *lac15* mutant has 59 % increase in soluble proanthocyanidin or condensed tannin in seeds [78]. Similar roles have been found for genes *BnTT10*, *PtLAC3*, and *ZmLAC3*, functioning in *B. napus*, *P. trichocarpa* and *Z. maize*, respectively [54, 77, 83].

Additionally, laccases are indicated to participate in plant response to environmental stresses, based on the findings that the expression of laccase was regulated by abiotic stresses [52, 65, 66]. Moreover, overexpression of a putative laccase gene from rice, *OsChI1*, increased the tolerance of transgenic Arabidopsis to drought and salinity stress [84]. The involvement of laccase in adversity stress response seems to be common, implying the potential of breeding energy crops on marginal lands, whereas further investigations are still needed to unravel the molecular mechanisms.

## Application of plant laccase in energy plant improvement

So far, only fungi and a few bacteria laccases have been utilized in industry [24, 37]. Fungi laccases with high redox potential are particularly useful than other ones in application, attributing to a broader range of substrates as well as the expanding utilization of mature laccase mediator system [85, 86]. Indeed, fungi laccases have been widely applied in pulp and paper, textile, food industries, organic synthesis, bioremediation and nanobiotechnology [23, 26].

Moreover, studies on enzyme immobilization have recently focused on laccase, mainly referred to fungi laccase, aiming to optimize good performance in industrial and large-scale applications. Various methods such as physical coupling (entrapment, encapsulation) and chemical interactions (adsorption, covalent binding and self-immobilization) have been developed [87–89]. Immobilization of laccase offers preserved enzymatic activity, improved storage and operational stability, and fine reusability for enzyme applications in the industrial process.

As for plant laccase, however, no example from industrial application has been reported yet. On the other hand, manipulation of laccase *in planta* may be a bold attempt for desirable lignin content and/or composition towards optimum plant biomass. This distinguishes largely from the way of removing lignin by ligninolytic enzymes (fungal laccase, together with manganese peroxidase, lignin peroxidase, and versatile peroxidase), since the former strategy takes aim at acquisition of optimal plant biomass fit directly for biofuel production.

The chemical composition, more specifically, the lignocellulosic matrix of plant species can affect the potential biofuel yield, according to a batch anaerobic digestion tests of 41 energy crops, in which 80 % of the sample variation on biofuel yield can be explained through lignin [90]. In wheat and rice, the H unit proportion of lignin has been revealed to be one of the dominant factors positively determining biomass digestibility [91]. Similarly in sweet sorghum, the G-monomer has been detected to have negative effects on biomass enzymatic digestibility and ethanol fermentation [92]. Hence, modification of lignin composition can be potentially employed for higher biomass saccharification in bioenergy crops. On the other hand, a compensatory mechanism between lignin and cellulose has been observed in transgenic aspen (*Populus tremuloides* Michx.) and the Arabidopsis *eli1* mutants, since repression of lignin biosynthesis promotes cellulose accumulation and reduced cellulose synthesis invokes lignification, respectively [93, 94]. From the perspective of energy utilization, increased cellulose content promises lower input but more output, providing directions and goals for energy plants improvement.

Previous studies have shown the inhibition of genes involved in monolignol biosynthesis, including phenylalanine ammonia lyase (*PAL*), cinnamate 4-hydroxylase (*C4H*), 4-coumarate:CoA ligase (*4CL*), caffeic acid (5-hydroxyconiferaldehyde) O-methyltransferase (*COMT*), and (hydroxy)cinnamoyl CoA reductase (*CCR*), could successfully alter lignin profiles. However, unfavorable phenotypes such as collapsed xylem and dwarf stature, decreased pollen viability, and altered leaf and flower morphology often accompanied the desirable changes [95]. Plant laccases catalyze the last step of monolignols oxidation and polymerization in lignin synthesis; furthermore, certain plant laccase members participated in specific lignin unit deposition, such as the Arabidopsis *LAC17* involved in the deposition of G lignin unit in the interfascicular fibers [60]. It is theoretically feasible to manipulate laccase gene expression for modifying lignin content and composition. The *BdLAC5*-misregulated *Bd4442* mutant in *B. distachyon* has been reported to show 10 % decreased Klason lignin content and modification of the S/G ratio, while the mutant showed higher saccharification efficiency. The results provided clear evidence that laccases are promising targets to alleviate the recalcitrance of grass lignocelluloses [55]. On the other hand, due to the functional redundancy of laccase members, indirect modification through the upstream miRNA engineering would be more efficient as one miRNA could target several laccases. It has been reported that transgenic Arabidopsis plants overexpressing miR397b have reduced lignin deposition as well as enlarged seeds and increased seed yield [6]. In rice, overexpression of *OsmiR397* enlarges grain size and promotes panicle branching, leading to an increase in overall grain yield of up to 25 % in a field trial [7]. Both of the studies indicated that regulation of laccase by miRNA have the potential for plant biomass engineering with less lignin and combined high yield properties.

To sum up, the application of plant laccase in biomass engineering through *in planta* manipulation of laccase/miRNA promises a bright development prospect, although no practice case has ever been reported in energy plant.

## Perspectives

So far, researches of plant laccase have been focused on cloning, bioinformatics analysis, and verification of the function of a single laccase gene. Besides, functional studies of plant laccase have been mainly carried out in model plants Arabidopsis and rice. Laccase is encoded by multigene family and has divergent functions. Therefore, the identification of laccase family members in different plant species and the evolutionary analysis should be essential for better understanding of their functions. Additionally, more information is required to determine the precise effect of laccase manipulation on biofuel production from lignocellulose, along with technical challenges in commercialization to be solved in the future.

In bioenergy production, tissue-specific promoters should be employed to achieve optimal laccase gene expression without negative effects on growth of transgenic energy plants, in order for maximized ethanol production and economic benefits. For example, it has been demonstrated in canola (*Brassica napus* L.) that the expression of *AtMYB32xs*::*IPT* could delay leaf senescence and improve seed yield, with no penalty on growth or phenology, which is clearly superior to transgenic plants carrying *IPT* genes driven by strong constitutive promoter, *SAG12* promoter, or *SARK* promoter [96].

Besides, the fact that certain members of plant laccases are involved in phenolic compounds oxidation imply the possibility of applying plant laccase for in vitro reaction or for industry in areas such as environmental pollution control, food industry, biosensors, textile industry, pharmaceutical industry, and in organic synthesis. Future studies on enzyme immobilization need pay attention to plant laccases as well.

The enhanced secretion of *ex planta* laccase may be of great value in phytoremediation of small organic pollutants. It has been reported in Arabidopsis that the overexpression of *LAC1*, a laccase gene specifically expressed in the roots of *Gossypium arboretum*, resulted in increased secretory laccase activity and enhanced resistance to several phenolic allelochemicals and 2,4,6-trichlorophenol (TCP) [97]. Therefore, *ex planta* phytoremediation, such as transgenic plants secreting extracellular laccases, can be another strategy for remediation of environmental contaminants.

## Conclusion

Plant laccase is an important oxidoreductase and shares the common molecular architecture and reaction mechanism with fungal laccase. However, it has lower redox potential and different pH requirement, which may partly account for its function in lignin biosynthesis, being contrary to the ligninolytic activity of fungal laccase. Plant laccase is encoded by multigene family, the tissue-specific expression of which may be regulated by transcription factors, miRNAs, and environmental stresses. Laccases are involved in various biological processes and plant response to environmental stresses. Most plant laccases are predicted to be localized in apoplast, in accordance with their functional involvement in lignin biosynthesis. Furthermore, laccase has been considered as a potential target for fine-tuning of plant lignin content and/or composition to alleviate the recalcitrance of lignocellulose, which promises a bright future for energy plant improvement in biofuel production.

## Additional file

**Additional file 1: Table S1.** Database accession numbers of laccase sequences for phylogenetic analysis.

## Abbreviations

4CL: 4-coumarate:CoA ligase
C4H: cinnamate 4-hydroxylase
CCR: (hydroxy)cinnamoyl CoA reductase
COMT: caffeic acid (5-hydroxyconiferaldehyde) O-methyltransferase
EPR: electronic paramagnetic resonance
MCOs: multicopper oxidases
pI: isoelectric point
PAL: phenylalanine ammonia lyase
TCP: 2,4,6-trichlorophenol
