# Supplementary material for: Lignin engineering through laccase modification: a promising field for energy plant improvement
Source: Biotechnol Biofuels. 2015 Sep 15;8:145. doi: 10.1186/s13068-015-0331-y (PMC4570640; doi:10.1186/s13068-015-0331-y)
Supplement: Supplementary file 1 — Additional file 1: Table S1. Database accession numbers of laccase sequences for phylogenetic analysis. [file 13068_2015_331_MOESM1_ESM.pdf]

Table 1 Database accession numbers of laccase sequences for phylogenetic analysis

Phylogenetic group		Laccase name	Accession number
**Plants**	*Arabidopsis thaliana*	AtLAC1	[TAIR: AT1G18140]
		AtLAC2	[TAIR: AT2G29130]
		AtLAC3	[TAIR: AT2G30210]
		AtLAC4	[TAIR: AT2G38080]
		AtLAC5	[TAIR: AT2G40370]
		AtLAC6	[TAIR: AT2G46570]
		AtLAC7	[TAIR: AT3G09220]
		AtLAC8	[TAIR: AT5G01040]
		AtLAC9	[TAIR: AT5G01050]
		AtLAC10	[TAIR: AT5G01190]
		AtLAC11	[TAIR: AT5G03260]
		AtLAC12	[TAIR: AT5G05390]
		AtLAC13	[TAIR: AT5G07130]
		AtLAC14	[TAIR: AT5G09360]
		AtLAC15	[TAIR: AT5G48100]
		AtLAC16	[TAIR: AT5G58910]
		AtLAC17	[TAIR: AT5G60020]
	Brachypodium distachyon	BdLAC5	[Phytozome: Bradi1g66720]
	Brassica napus	BnTT10-1	[Genbank: AEK27149]
	Gossypium arboreum	GaLAC1	[Genbank: AAR83118]
	Populus trichocarpa	PtLAC3	[EMBL: CAA74103]
		PtLAC90	[EMBL: CAA74104]
		PtLAC110	[EMBL: CAA74105]
	Saccharum officinarum	SofLAC	[SUCEST: SCUTST3084C11.g]
	Zea mays	ZmLAC1	[Genbank: AAX83112]
		ZmLAC2	[EMBL: CAJ30498]
		ZmLAC3	[EMBL: CAJ30499]
		ZmLAC4	[EMBL: CAJ30500]
		ZmLAC5	[EMBL: CAJ30497]
**Fungi**	*Botrytis cinerea*	BcLcc1	[Genbank: AAK77952]
		BcLcc2	[Genbank: AAK77953]
	Colletotrichum lagenarium	ClLac1	[Genbank: BAB32575]
	Coprinopsis cinerea	CcLcc1	[Genbank: AAD30964]
		CcLcc2	[Genbank: AAD30965]
		CcLcc3	[Genbank: AAD30966]
	Cryphonectria parasitica	CpLac1	[Genbank: AAA09235]
	Melanocarpus albomyces	MaLac1	[EMBL: CAE00180]
	Neurospora crassa	NcLac1	[Genbank: EAA27703]
	Podospora anserina	PaLac2	[EMBL: CAA70061]
	Polyporus ciliatus	PcLcc3-2	[Genbank: AAG09230]
	Pycnoporus cinnabarinus	PcLac1	[Genbank: AAG13724]

	Trametes versicolor	TveLac 1	[Genbank: BAD98305]
		TveLac 2	[Genbank: BAD98306]
		TveLac 3	[Genbank: BAD98307]
		TveLac 4	[Genbank: BAD98308]
	Trametes villosa	TviLcc1	[Genbank: AAC41686]
		TviLcc2	[Genbank: AAC41687]
		TviLcc3	[Genbank: AAB47733]
		TviLcc4	[Genbank: AAB47734]
		TviLcc5	[Genbank: AAB47735]
**Insects**	*Aedes aegypti*	AaLac1	[Genbank: AAY29698]
		AaLac2	[Genbank: AAY32604]
	Bombyx mori	BmLac2	[Genbank: BAG70891]
	Culex pipiens pallens	CppLac2	[Genbank: ACG63789]
	Manduca sexta	MsLac1	[Genbank: AAN17506]
		MsLac2	[Genbank: AAN17507]
	Megacopta punctatissima	MpLac2	[Genbank: BAJ83488]
	Monochamus alternatus	MaLac2	[Genbank: ABU68466]
	Nephotettix cincticeps	NcLac1S	[Genbank: BAJ06131]
		NcLac1G	[Genbank: BAJ06132]
		NcLac2	[Genbank: BAJ06133]
	Nilaparvata lugens	NlMCO2	[Genbank: AKN21380]
	Pimpla hypochondriaca	PhLac1	[EMBL: CAD20461
	Reticulitermes flavipes	RfLacA	[Genbank: ACX54558]
		RfLacB	[Genbank: ACX54560]
	Riptortus pedestris	RpLac2	[Genbank: BAJ83487]
	Tribolium castaneum	TcLac1	[Genbank: AAX84206]
		TcLac2A	[Genbank: AAX84202]
		TcLac2B	[Genbank: AAX84203]
**Bacteria**	*Bacillus subtilis*	BsCotA	[EMBL:CAB12449]
	Bacillus halodurans	BhLbh1	[Genbank: AAP57087]
	Escherichia coli	EcCueO	[Genbank: AAC73234]
	Marinomonas mediterranea	MmPpoA	[Genbank: AAF75831]
	Oceanobacillus iheyensis	OiCotA	[Genbank: BAC13302]
	Pseudomonas putida	PpCumA	[Genbank:AAD24211]
	Streptomyces griseus	SgEpoA	[Genbank: BAB64332]
	Streptomyces lavendulae	SlSTSL	[Genbank: BAC16804]
